# Bioinformatics and Expression Analysis of IDA-Like Genes Reveal Their Potential Functions in Flower Abscission and Stress Response in Tobacco (*Nicotiana tabacum* L.)

**DOI:** 10.3389/fgene.2021.670794

**Published:** 2021-04-27

**Authors:** Cun Guo, Qi Wang, Zhiyuan Li, Jinhao Sun, Zenglin Zhang, Xiaoxu Li, Yongfeng Guo

**Affiliations:** ^1^Tobacco Research Institute, Chinese Academy of Agricultural Sciences, Qingdao, China; ^2^Graduate School of Chinese Academy of Agricultural Sciences, Beijing, China; ^3^Technology Center, China Tobacco Hunan Industrial Co., Ltd., Changsha, China

**Keywords:** flower abscission, IDA peptide, IDL, tobacco, *cis*-element, abiotic stresses

## Abstract

The inflorescence deficient in abscission-like (*IDL*) genes have been shown to play critical roles in floral organ abscission, lateral root formation and various stress responses in *Arabidopsis*. The *IDL* gene family has been characterized in a number of plant species, while limited information is available about *IDL* genes of tobacco. In the current study, 15 NtIDL members were identified in the tobacco genome, and were classified into six groups together with IDL members from other species. Evolution analysis suggested that the NtIDL members form group VI might have originated from duplication events. Notably, NtIDL06 shared high similarities with AtIDA in the EPIP sequence, and its encoding gene was highly expressed in the abscission zone of flowers at late developmental stages, implying that NtIDL06 might regulate tobacco flower abscission. In addition, the results from *cis*-elements analysis of promoters and expression after stress treatments suggested that *NtIDL* members might be involved in various stress responses of tobacco. The results from this study provide information for further functional analysis related to flower abscission and stress responses of *NtIDL* genes.

## Introduction

Abscission is a highly coordinated cell separation process in plants. From an evolutionary perspective, active abscission is advantageous in many aspects for plants, such as dispersal, propagation, pollination and defense (Lewis et al., 2006). It allows parent plants to abandon damaged organs no longer needed (Reichardt et al., 2020). A prerequisite for abscission to transpire is the presence of an abscission zone, which is composed of small densely cytoplasmic cells that respond to abscission signals (Patterson, 2001; Liljegren, 2012; Gubert et al., 2014). Also, abscission can be triggered by multiple factors, including seasonal changes, pathogen attack, abiotic stresses, and hormones (Patharkar and Walker, 2019). Abscisic acid (ABA) and Methyl Jasmonate (MeJA) were reported to accelerate organ abscission, while auxin and brassinosteroids were negative regulators of shedding (Hartmond et al., 2000; Chandler, 2011; Marciniak et al., 2018; Wilmowicz et al., 2018; Mesejo et al., 2021).

In *Arabidopsis*, flower organ abscission is dependent on the function of a small peptide that is released from the IDA (inflorescence deficient in abscission) precursor protein (Reichardt et al., 2020). The IDA proprotein is composed of 77 amino acids including an N-terminal signal peptide and a C-terminal EPIP domain (FGYLPKGVPIPPSA PSKRHNSFVNSLPH). The EPIP domain (extended PIP) was confirmed to be the main functional domain of the IDA protein (Stenvik et al., 2008b). The abscission of *ida* mutant flower organs failed to appear, while the flowers fall off prematurely in the plants overexpressing the *IDA* gene (Stenvik et al., 2008b; Kumpf et al., 2013; Liu et al., 2013). It has been shown that the IDA peptide functions as a ligand of the receptor-like kinases HAESA and HAESA-LIKE2 (HAE/HSL2), which dominates flower abscission. The IDA-HAE/HSL2 pathway was shown to activate downstream mitogen-activated protein (MAP) kinase cascades, which regulate the expression of hydrolytic and cell wall-modifying enzymes (Jinn et al., 2000; Stenvik et al., 2008a; Kumpf et al., 2013; Meng et al., 2016). Also, somatic embryogenesis receptor-like kinase (SEKR) was reported to act as a co-receptor for IDA with HAE/HSL2 to transmit the abscission signal (Santiago et al., 2016; Patharkar and Walker, 2018).

Except for being involved in flower abscission, the IDA-HAE/HSL2 signaling module was reported to be important for lateral root emergence (Matsubayashi and Sakagami, 2006; Kumpf et al., 2013; Shi et al., 2018; Zhang et al., 2020). Several *IDL* genes was recently reported to be involved in responding to multiple stresses in *Arabidopsis* (Vie et al., 2017; Wang et al., 2017). *AtIDL6* expression was up-regulated by *Pseudomonas syringae* pv. tomato (*Pst*) DC3000 infection. Overexpression and knockdown lines of *AtIDL6* showed decreased and increased resistance to *Pst* DC3000 in *Arabidopsis*, respectively. Moreover, *AtIDL6* and *AtIDL7* were suggested to be induced rapidly by various stresses as negative modulators of stress-induced reactive oxygen species (ROS) signaling (Vie et al., 2017; Wang et al., 2017).

The regulation of flower abscission by genes encoding IDL peptides seems to be conserved in plants (Tranbarger et al., 2019; Schuster and Van Der Hoorn, 2020). For instance, the *SlIDA1* genes were closely related to drought-induced tomato flower drop (Tucker and Yang, 2012). In *Citrus*, five *CitIDA* genes were identified, and overexpression of *CitIDA3* gene complemented the abscission deficiency of the *ida* mutant in *Arabidopsis* (Estornell et al., 2015). Besides, *LcIDL1* was identified as a homologous gene of *AtIDA* from the litchi genome, and it was reported to play a role in regulating the shedding of floral organs in *Arabidopsis* (Ying et al., 2016). Interestingly, *IDL* genes were also found in root-knot nematodes (*Meloidogyne incognita*), and exogenous treatments of *ida* mutant plants with synthetic MiIDL1 peptides caused petals to abscise in *Arabidopsis* (Kim et al., 2018).

Tobacco (*Nicotiana tabacum* L.) is one of the most important non-food crops and has been widely used as a model plant for analyzing gene function (Li et al., 2021). A recent genome-wide study revealed a range of flower-related genes in tobacco, such as the *MADS-box* gene family (Bai et al., 2019). While study of *IDL* genes in the control of flower abscission is limited in *N. tabacum*. Here, we report the identification of *IDL* and *HAE-Like* genes from the tobacco genome. Expression analysis showed that individual *NtIDL* members and *HAE-Like* genes were highly expressed in the abscission zone of the late stages of flower development. Furthermore, the results from expression analysis also suggested that *NtIDL* genes might be involved in stress responses in tobacco.

## Materials and Methods

### Identification and Sequence Analysis of NtIDL and NtHAE Members

The protein sequences of *Arabidopsis* IDA and IDL1-8 were downloaded from TAIR (Lamesch et al., 2012) and used as probe sequences to search the tobacco genome database (Edwards et al., 2017) with the E-value cutoff of 0.01. Newly identified genes were named according to the information of chromosomes and scaffold numerically. Similarly, the protein sequences of *Arabidopsis* HAE and HSL2 were used as queries to carry out BLASTP searches against the tobacco genome database under the E-value cutoff of 0.001. Newly identified *NtHAE-Like* genes were named according to the evolutionary analysis. Each sequence was submitted to ProtParam^1^ to predict isoelectric point and molecular weight.

### Multiple Sequence Alignment and Phylogenetic Analysis

Multiple sequence alignment of NtIDL and reported IDLs from other species was performed using MAFFT, with their full-length amino acid sequences under default settings (Katoh and Standley, 2013). Base on the sequence alignment results, MEGA X was used to generate a neighbor-joining (NJ) phylogenetic tree (Kumar et al., 2016). The EPIP sequences of all members were extracted for multiple sequence alignment and visualized together with the results of evolutionary analysis.

### Analysis of *Cis*-Elements in the Promoter of *NtIDL* Members

To assess the *cis*-elements of the *NtIDL* promoters, 2000 base pairs of promoter regions upstream of the start codon of the *NtIDL* genes were extracted, according to a previous report (Cao et al., 2016). The PlantCARE database was engaged for *cis*-elements investigation, and the results were visualized by the TBtools (Lescot et al., 2002; Chen et al., 2020).

### Plant Growth Conditions

Seeds of tobacco cultivar K326 were germinated and cultured using a floating seedling production system under normal conditions (28°C, 14 h light, 10 h dark). The tissues (root, stem, shoot, leaf and flower) and abscission zones of flowers at different developing stages were collected to analyze *NtIDL* gene expression. For hormones and salt treatments, tobacco seedlings were germinated on MS medium in a light incubator at 25°C for 2 weeks and treated with 50 μM ABA, 100 μM MeJA or 150 mM NaCl following a previous report (Li et al., 2019). For low/high temperature and drought treatments, the seedlings were placed in a growth chamber at 4°C/37°C or placed on filter paper. For wounding treatment, a sterile surgical blade was used to mechanically damage the third leaf of tobacco seedlings along the veins. Whole seedlings were collected at 0, 3, and 6 h after treatments, frozen in liquid nitrogen and transferred to −80°C for storage. Triple biological replicates were performed for each sample.

### RNA Extraction and qRT-PCR

Total RNA of all samples was extracted following the instructions of Ultrapure RNA Kit (cwbiotech, Beijing, China). The quality and quantity of the isolated total RNA were determined by NanoDrop (Thermo Scientific^TM^) and gel blot analysis. cDNA synthesis was performed using same amount of RNA according to the directions of the kit (R323-01, Vazyme, Nanjing, China). The tobacco ribosomal protein gene *L25* (GenBank No. L18908) was adopted as the control. qRT-PCR was performed on Roche LightCycler^®^ 480 in a 20 μL reaction with SYBR (TaKaRa, Shiga, Japan) 10 μL, 10 mM forward primer 0.4 μL, 10 mM reverse primer 0.4 μL, and diluted cDNA 0.2 μL. Three independent experiments were carried out with three technical replicates, and the average value was taken for analysis based on the 2^–ΔΔCt^ method. The primer pairs used are listed in Supplementary Table 1.

## Results

### Identification of *IDL* Family Genes in Tobacco (*Nicotiana tabacum* L.)

To identify IDL proteins in the tobacco proteome, the *Arabidopsis* IDA and IDL1-8 proteins were employed as queries to search against the local tobacco proteome database using Blastp. After manually removing repeated sequences, a total of 15 *IDL* genes were obtained from tobacco proteome. For consistency, newly identified IDL family members were named NtIDL01-NtIDL15 in the order of chromosome and scaffold. The detailed information of gene localization and protein characteristics were listed in Table 1. Amino acid length analysis showed that tobacco IDL family members ranged from 73 aa (NtIDL07) to 126 aa (NtIDL10). Their theoretical isoelectric points were from 5.19 (NtIDL13) to 11.17 (NtIDL15), and the molecular weight ranged from 7,723.83 Da (NtIDL07) to 14,120.9 Da (NtIDL10).

**TABLE 1:** IDA-Like gene family members in tobacco*.

**Genes**	**Access number**	**Chr/Scf**	**5′ End**	**3′ End**	**AA**	**pI**	**MW**	**Group**
*NtIDL01*	Nitab4.5_0000788g0070.1	Nt02	82,995,999	82,996,316	105	10.44	11,636.68	IV
*NtIDL02*	Nitab4.5_0002578g0020.1	Nt03	32,951,224	32,951,508	94	9.86	9,882.36	VI
*NtIDL03*	Nitab4.5_0001984g0090.1	Nt12	100,853,473	100,853,736	87	8.11	9,236.66	VI
*NtIDL04*	Nitab4.5_0001027g0160.1	Nt13	22,578,392	22,578,655	87	7.95	9,246.7	VI
*NtIDL05*	Nitab4.5_0000419g0050.1	Nt14	89,956,424	89,956,687	87	7.96	9,224.67	VI
*NtIDL06*	Nitab4.5_0000027g0380.1	Nt24	109,387,139	109,387,381	80	9.13	8,945.4	III
*NtIDL07*	Nitab4.5_0001185g0060.1	Nitab4.5_0001185	437,880	484,907	73	9.52	7,723.83	VI
*NtIDL08*	Nitab4.5_0003346g0040.1	Nitab4.5_0003346	40,233	49,492	116	7.23	12,812.6	VI
*NtIDL09*	Nitab4.5_0004688g0060.1	Nitab4.5_0004688	91,864	92,118	84	9.34	9,350.81	III
*NtIDL10*	Nitab4.5_0004965g0010.1	Nitab4.5_0004965	62,139	67,203	126	6.1	14,120.94	VI
*NtIDL11*	Nitab4.5_0005426g0020.1	Nitab4.5_0005426	169,912	170,157	81	10.38	8,818.35	II
*NtIDL12*	Nitab4.5_0005633g0020.1	Nitab4.5_0005633	157,769	159,007	73	5.19	7,972.8	VI
*NtIDL13*	Nitab4.5_0007980g0010.1	Nitab4.5_0007980	41,307	41,585	92	6.71	9,757.18	VI
*NtIDL14*	Nitab4.5_0008298g0010.1	Nitab4.5_0008298	85,973	86,302	109	9.51	12,002.92	IV
*NtIDL15*	Nitab4.5_0012260g0070.1	Nitab4.5_0012260	35,972	36,513	100	11.17	10,920.81	II

*Chr, chromosome; Scf, scaffolds; AA, the number of Amino acids; pI, isoelectric points; MW, molecular weights.

### Multiple Sequence Alignment and Evolution Analysis of IDL Family Members

**FIGURE 1:**
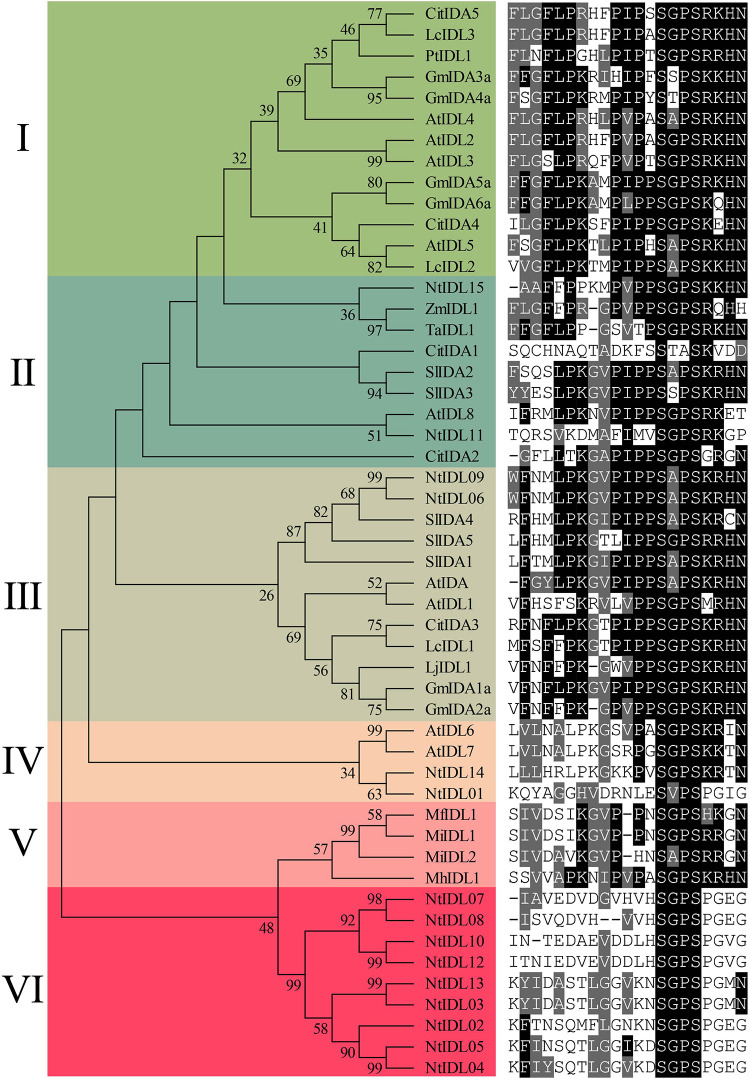
Phylogenetic analysis of NtIDL family members. *Citrus* (Cit), *Litchi chinensis* (Lc), *Populus* (Pt), *Glycine max* (Gm), *Arabidopsis thaliana* (At), *Solanum lycopersicum* (Sl), *Nicotiana tabacum* (Nt), *Zea mays* (Zm), *Triticum aestivum* (Ta), southern root-knot nematode (*Meloidogyne incognita*, Mi), northern root-knot nematode (*Meloidogyne. hapla*, Mh), and peach root-knot nematode (*Meloidogyne. floridensis*, Mf). On the right is the conservative EPIP sequences alignment.

To explore the conservation of tobacco IDLs during evolution, a number of representatives IDL sequences from previous studies (Tucker and Yang, 2012; Estornell et al., 2015; Ying et al., 2016; Kim et al., 2018; Liu et al., 2018) together with the newly identified NtIDL members were subjected to multiple sequence alignments using MAFFT, and a neighbor-joining tree was generated by MEGA X. Thereafter, the EPIP domains of all the IDL members were extracted and displayed together with the results of evolutionary analysis (Figure 1).

As a result, all the IDL proteins were classed into six groups, namely I-VI, based on the topology of the phylogenetic tree. Most of the groups contained two or more tobacco IDL members. For example, NtIDL15 and NtIDL11 are in group II, where they were clustered with ZmIDL1, TaIDL1, and AtIDL8. NtIDL06 and NtIDL09 belong to group III together with AtIDA and AtIDL1, and they both share 85.7% similarities with AtIDA in amino acid EPIP sequence (Figure 1). In group IV, NtIDL01 and NtIDL14 were clustered together with AtIDL6 and AtIDL7. The remaining NtIDL members are all in group VI, which is unique to tobacco. No tobacco IDL member was grouped in Group I. Group V only contains IDL members of root-knot nematodes, implying a unique evolution path shared by the nematode IDLs.

### Analysis of *Cis*-Elements in the Promoters of *NtIDLs*

**FIGURE 2:**
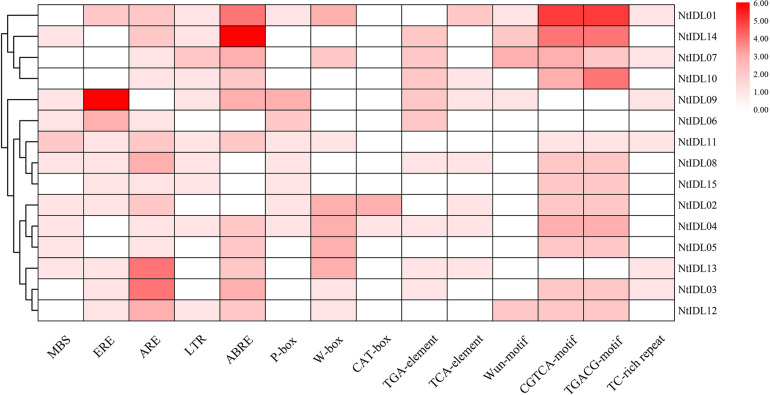
Regulatory elements in the promoter regions of *NtIDL* gene promoters. The color represents the numbers of *cis*-element in *NtIDL* gene promoters. MBS (MYB binding site involved in drought-inducibility), ERE (ethylene-responsive *cis*-acting), ARE (anaerobic induction element), LTR (low-temperature-responsive element), ABRE (*cis-*acting element involved in the abscisic acid responsiveness), P-box (gibberellin-responsive element), W-box (WRKY binding site), CAT-box (*cis-*acting regulatory element related to meristem expression), TGA-element (auxin-responsive element), TCA-element (*cis-*acting element involved in salicylic acid responsiveness), Wun-motif (wound-responsive element), CGTCA-motif (*cis-*acting regulatory element involved in the MeJA-responsiveness), TGACG-motif (*cis-*acting regulatory element involved in the MeJA-responsiveness), and TC-rich repeats (involved in defense and stress responsiveness).

The study of *cis*-elements could provide clues about regulatory pathways of gene expression. Therefore, the promoter regions of 15 *IDL* genes in tobacco were analyzed using the PlantCARE Online toolboxes (Lescot et al., 2002). In general, various *cis*-elements were identified in the tobacco *IDL* gene promoters. 14 *cis*-elements involved in different hormone response, developmental process and stress response were selected for further analysis (Figure 2). As a result, 11 *NtIDL* promoters contain ABRE *cis*-acting elements involved in ABA responsiveness. Among them, the *NtIDL14* promoter contains 6 ABRE *cis*-elements. Both CGTCA-motif and TGACG-motif were related to MeJA responsive. 12 *NtIDL* promoters were found to possess these two kinds of elements. Also, ethylene-responsive *cis*-element (ERE), salicylic acid (TCA-element), gibberellin (P-box) and auxin (TGA-element) were identified on *NtIDL* promoters. These results suggest that hormones may play important roles in regulating *NtIDL* expression.

Notably, stress-responsive elements including MBS (MYB binding site involved in drought-inducibility), TC-rich repeats (involved in defense and stress responsiveness), LTR (low-temperature-responsive element), WUN-motif (wound-responsive element), and ARE (anaerobic induction element) were founded to be abundant in the promoter regions of a large number of *NtIDL* genes. Interestingly, nine *NtIDL* genes were predicted to contain W-box *cis*-elements, which act as the WRKY transcription factors’ binding site, implying certain WRKY transcription factors might regulate these *NtIDL* genes. Overall, *NtIDL* promoters possess abundant stress-related *cis*-elements, suggesting that tobacco *IDL* genes might be regulated by multiple stresses.

### Expression Profiles of *IDL* and *HAE-Like* Genes of Tobacco

**FIGURE 3:**
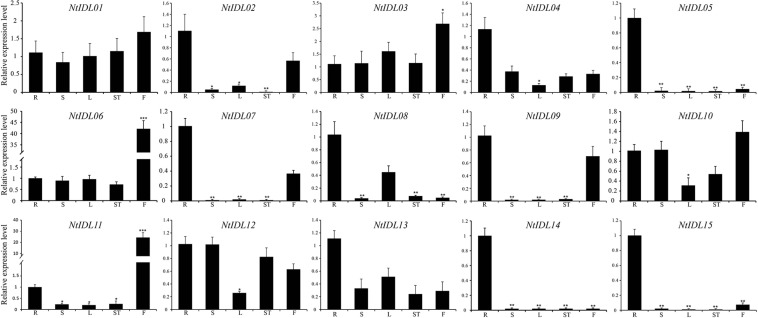
The expression patterns of *NtIDL* genes in selected tissues. “R,” “S,” “L,” “ST,” and “F” mean roots, stems, leaves, shoots and flowers, respectively. Their expression was calculated as folds relative to the expression level of the roots to confirm the tissue specificity. All expression levels were calculated through the 2^–ΔΔCt^ method. The data were means ± SD from three independent replications. *p < 0.05, **p < 0.01, and ***p < 0.001 (*t*-test).

To explore the expression patterns of *NtIDL* members, different tissues from tobacco seedlings were collected and analyzed, including roots, stems, leaves, shoots and flowers. The results showed several *NtIDL* genes were detected to be expressed in all these tissues (Figure 3), such as *NtIDL01, NtIDL03*, *NtIDL06*, *NtIDL10*, and *NtIDL12*. In comparison, transcripts of some other *NtIDL* genes were presented at high levels in specific tissues. For instance, *NtIDL02* and *NtIDL09* were highly expressed in roots and flowers. Expression of *NtIDL05*, *NtIDL14*, and *NtIDL15* was significantly higher in roots that in the other tissues. It was worth noting that *NtIDL06* and *NtIDL11* were highly expressed in flowers, suggesting that both *NtIDL06* and *NtIDL11* might play significant roles in flower development of tobacco.

**FIGURE 4:**
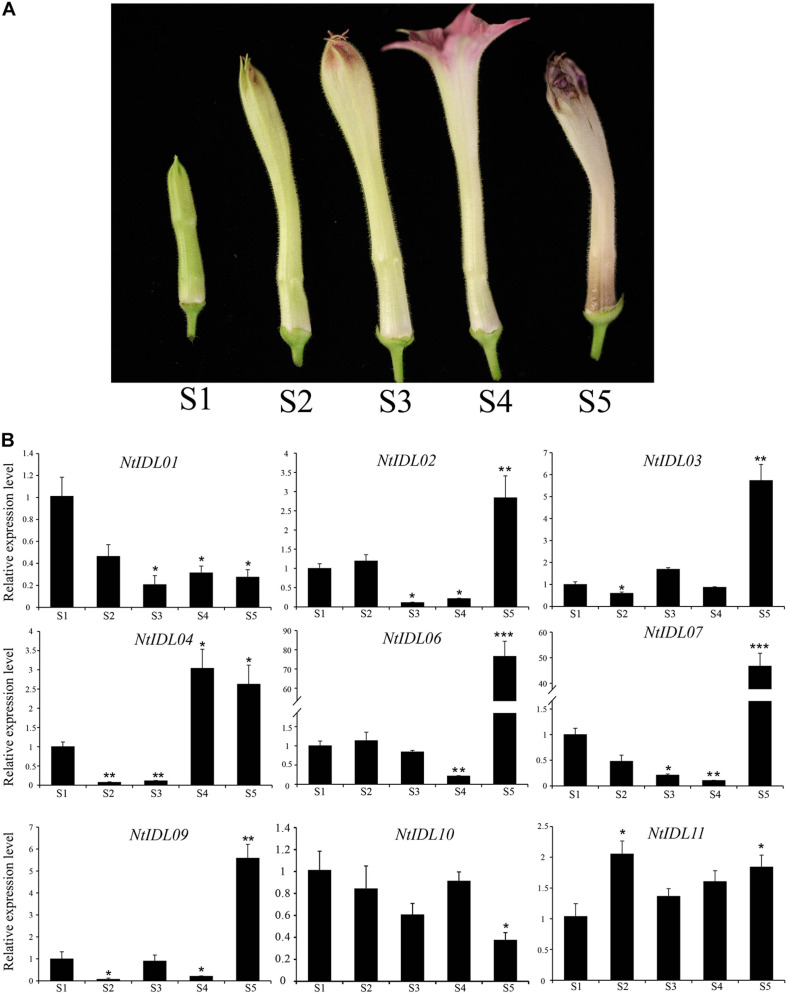
The expression patterns of *NtIDL* genes in abscission zones during flower development. **(A)**. Five stages of tobacco flower development. “S1–S5” means “Stage 1 to Stage 5 of flower development” **(B)**. The expression patterns of selected *NtIDL* genes in the abscission zone during the flower development. All expression levels were calculated through the 2^–ΔΔCt^ method. The data were means ± SD from three independent replications. *p < 0.05, **p < 0.01, and ***p < 0.001 (*t*-test).

In order to explore potential roles of NtIDLs in flower abscission, representative *NtIDL* genes were selected to perform expression analysis in abscission zones during floral organ development. Flower development was divided into five stages as shown in Figure 4A. As a result (Figure 4B), *NtIDL* genes exhibited various expression patterns in the abscission zone during flower development. The expression of *NtIDL01* was down-regulated during the development of flowers. In contrast, *NtIDL02*, *NtIDL03*, *NtIDL04*, and *NtIDL09* were up-regulated during flower development. Especially, *NtIDL06* and *NtIDL07* showed significantly higher expression at the last stage of flower development, implying that they might be closely related to the regulation of flower abscission.

In addition, the expression patterns of putative receptors of the NtIDL peptides in tobacco, *NtHAEa*, *NtHAEb*, *NtHSL2a*, and *NtHSL2b*, were also analyzed (Supplementary Figure 2). The results showed that the receptor-encoding genes were expressed in all the tested tissues. All of them showed high-level expression in flowers. Interestingly, the *HAE-Like* genes of tobacco were highly expressed in the abscission zone of at late stages of flower development, which is similar to the expression pattern of some *NtIDL* genes, including *NtIDL06*, *NtIDL07*, and *NtIDL09*.

### Expression of *NtIDL* Genes Under Multiple Abiotic Stresses

**FIGURE 5:**
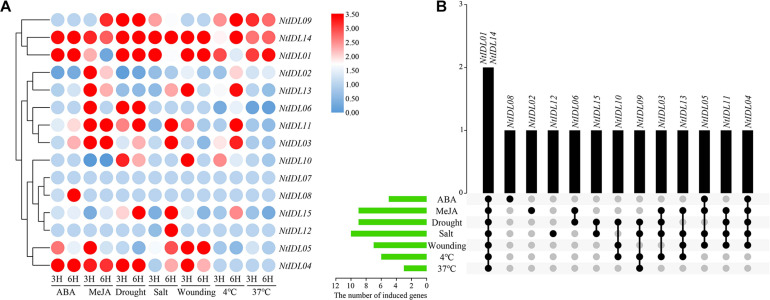
The expression patterns of *NtIDL* genes in tobacco seedlings under multiple abiotic stress treatments. **(A)** The relative expression ratios of abiotic stress treatments, “3H” and “6H” represent 3 and 6 h after stress treatments, respectively. Expression levels were calculated through the 2^–ΔΔCt^ method and normalized relative to gene expression in control plants. Expression levels relative to control plants are given next to the color scale. Red, white and blue represents the induction (values over 1), invariance (values close to 1) and inhibition (values under 1) of genes related to the control treatment, respectively. **(B)** The summarized information of the stress-induced *NtIDL* genes. Black dots indicate *NtIDL* genes responsive to stress treatments and gray dots indicate *NtIDL* genes that did not respond to the stress treatments.

Promoter regions of *NtIDLs* contain various *cis*-elements that are responsive to hormones and stresses. Therefore, qRT-PCR was performed to study the expression changes of *NtIDLs* under different abiotic stress treatments, including ABA, MeJA, drought, salt, wounding, and low/high temperature. All the 15 *NtIDL* genes were tested and showed complex expression patterns under various abiotic stress treatments (Figure 5A). As a result, *NtIDL01*, *NtIDL14*, and *NtIDL04* were up-regulated under ABA treatment. In contrast, the transcription level of *NtIDL02* was down-regulated by ABA. Interestingly, *NtIDL05* was up-regulated after 3 h but not after 6 h of ABA treatment, while *NtIDL08* showed high expression only after 6 h of ABA treatment. For MeJA treatment, nine genes were up-regulated, including *NtIDL04* and *NtIDL14*, while *NtIDL10* and *NtIDL15* were down-regulated by MeJA.

In addition, a number of *NtIDL* genes also responded to abiotic stress treatments. Some of the *NtIDL* genes were induced under multiple stresses (Figure 5B). *NtIDL01* and *NtIDL14* were induced by all the seven stress treatments, and *NtIDL04* was induced by all the stresses except for the low/high-temperature treatments. *NtIDL03*, *NtIDL05*, *NtIDL09*, *NtIDL11*, and *NtIDL13* were up-regulated by four different stress treatments. Three of the *NtIDLs*, on the other hand, were only induced by one specific stress treatments: *NtIDL08* was only induced by ABA treatment, *NtIDL02* was only induced by MeJA, and *NtIDL12* was only induced by salt treatment. Notably, *NtIDL07* has not been detected to be induced by any treatment in this study. Among the different stress treatments, salt treatment could induce the most *NtIDL* genes (10), while high-temperature treatment (37°C) only induced three *NtIDL* genes.

## Discussion

The IDL peptides have been shown to play critical roles in floral organ abscission, lateral root formation and various stress responses (Jinn et al., 2000; Liljegren, 2012; Wang et al., 2020). Systematic identification and analysis of the *IDL* gene family have been performed in many crops. However, there is less information on the *IDL* genes of tobacco. In the current study, the identification, evolution, classification, and expression profile were performed to study IDL members in tobacco.

A total of 15 NtIDL members were identified form the tobacco genome. These NtIDL members were divided into six groups with IDL members from other plant species (Figure 1). Notably, in group VI, all of the IDL members were from tobacco, suggesting that these NtIDL members might be originated from duplication events. Due to the fact that we were not able to map most of the *NtIDL* genes to the tobacco chromosomes (Table 1), the related duplication events could not be analyzed yet in the current study. Results from multiple sequence alignment analysis indicated that the EPIP sequences of IDL family members have high similarities, suggesting that IDL members might have maintained conserved functions during evolution. Interestingly, IDL proteins were also found in the root-knot nematode (*M. incognita*) genome, and they were shown to be involved in the regulation of plant root development (Kim et al., 2018). In this study, the root-knot nematode IDL members were analyzed and clustered together with NtIDL members of group VI. Root-knot nematode diseases caused by *M. incognita* are one of the most destructive diseases in tobacco production (Li et al., 2018). Most of the *NtIDLs* in group VI, including *NtIDL02*, *NtIDL04*, *NtIDL05*, *NtIDL07*, and *NtIDL13*, were highly expressed in roots (Figure 3). Whether the nematode-encoded IDL peptides play a role in the establishment of the infection of root-knot nematodes on tobacco, remains to be elucidated.

Previous studies indicated that some *IDL* genes encode small peptides that mediate in plants’ responses to abiotic stresses. In group III, *NtIDL09* was up-regulated under high temperature, salt, and drought treatments (Figure 5A). While *NtIDL06*, also in group III, was down-regulated by high-temperature treatment (Figure 5A). This result implies that functional divergence might have occurred within this group. In group IV, NtIDL01 and NtIDL14 were clustered with AtIDL6 and AtIDL7 (Figure 1). These two *Arabidopsis* members have been reported to be induced rapidly by various stresses (Vie et al., 2017). Interestingly, abundant stress-related *cis*-acting elements were identified in the promoter regions of *NtIDL01* and *NtIDL14* (Figure 2), and both of them were induced by multiple stresses (Figure 5A), suggesting their potential functions in multiple stress responses.

The *cis*-elements analysis showed that *NtIDL* members contained rich response-hormone *cis*-elements on their promoters (Figure 2), which suggested that hormones might be involved in the transcriptional regulation of *NtIDL* genes. Phytohormones ABA and MeJA have been reported to accelerate flower abscission in plants (Hartmond et al., 2000; Patharkar and Walker, 2019). Moreover, *NtIDL01*, *NtIDL14*, *NtIDL08*, *NtIDL04*, and *NtIDL05* were found to be up-regulated under ABA treatment (Figure 5A). Also, nine *NtIDL* genes, including *NtIDL02* and *NtIDL06*, were induced by MeJA treatment. Taken these results, these NtIDL members may confer flower abscission though the ABA and MeJA signaling pathways.

In *Arabidopsis*, overexpression of the *AtIDA* gene could rescue the deficiency in flower abscission of the *ida* mutant. Notably, NtIDL06 and NtIDL09 were clustered together with AtIDA and AtIDL1 in group III. IDL members from other plant species in this group were also reported to regulate flower abscission, including SlIDA1 (Tucker and Yang, 2012), CitIDA3 (Estornell et al., 2015), LcIDL1 (Ying et al., 2016), and GmIDA1a (Tucker and Yang, 2012; Figure 1). Moreover, NtIDL06 shared high similarities with AtIDA in EPIP sequences (Figure 1). The qRT-PCR results indicated that *NtIDL06* was highly expressed in the abscission zone at the last stage of flower development (Figure 4). Moreover, four *NtHAE-Like* genes identified in this study were detected to show similar expression patterns with *NtIDL06* (Supplementary Figure 2). Those genes with high expression levels in the abscission zone at late stages of flower development might be related to cell wall remodeling and abscission of flowers. It is worth mentioning that *NtIDL06* was induced by MeJA treatment (Figure 5A), and MeJA was a positive regulator of flower abscission. Combining these results, *NtIDL06* might be involved in tobacco flower abscission.

## Conclusion

Systematic investigation was adopted to identify 15 *NtIDL* genes in the tobacco genome. The results from expression analysis in different tissues and under various of stress treatments suggested that the tobacco *IDL* genes might play multiple roles in various biological processes. A number of NtIDLs were identified with potential functions in stress responses. Notably, as the closest homolog of AtIDA, *NtIDL06* and its putative receptors were highly expressed in the abscission zone at the last stage of flower development, suggesting that NtIDL06 might be involved in the natural process of corolla abscission. The results from this study provide insights for further exploring the biological functions of tobacco *IDL* genes.

## Data Availability Statement

The original contributions presented in the study are included in the article/Supplementary Material, further inquiries can be directed to the corresponding author/s.

## Author Contributions

YG and XL conceived this research and designed the experiments. CG and QW conducted the research and drafted the manuscript. ZL, JS, and ZZ assisted in data collection and participated in drafting the manuscript. All authors contributed to the article and approved the submitted version.

## Conflict of Interest

XL was employed by China Tobacco Hunan Industrial Co., Ltd. The remaining authors declare that the research was conducted in the absence of any commercial or financial relationships that could be construed as a potential conflict of interest. The handling editor declared a past co-authorship with one of the authors XL.

## Footnotes

1 http://au.expasy.org/tools/protparam.html

## Supplementary Materials

Supplementary Figure 1 Sequence alignment of HAE-Like proteins from tobacco and *Arabidopsis*. The black background indicates that the amino acid similarity is 100%, and the gray indicates that the amino acid similarity is ranged from 60 to 90%.

Supplementary Figure 2 The expression pattern of *NtHAE-Like* genes in selected tissues **(A)** and in abscission zone during the flower development **(B)**. Panel **(A)**: “R,” “S,” “L,” “ST,” and “F” mean roots, stems, leaves, shoots and flowers, respectively. Their expressions were calculated as folds relative to the expression level of the roots to confirm the tissue specificity. Panel **(B)**: “S1–S5” means “Stage 1 to Stage 5 of flower development.” All expression levels were calculated through the 2^–ΔΔCt^ method. The data were means ± SD from three independent replications. **p* < 0.05, ***p* < 0.01, (*t*-test).

Supplementary Table 1 The qRT-PCR primers used in this study.

Supplementary Table 2 HAE-Like gene family members in tobacco.

## References

[B1] Bai G. Yang D.-H. Cao P. Yao H. Zhang Y. Chen X. (2019). Genome-wide identification, gene structure and expression analysis of the MADS-Box gene family indicate their function in the development of tobacco (*Nicotiana tabacum L.*). *Int. J. Mol. Sci.* 20:5043. 10.3390/ijms20205043 31614589 PMC6829366

[B2] Cao Y. Han Y. Li D. Lin Y. Cai Y. (2016). MYB transcription factors in chinese pear (Pyrus bretschneideri Rehd.): genome-wide identification, classification, and expression profiling during fruit development. *Front. Plant Sci.* 7:577. 10.3389/fpls.2016.00577 27200050 PMC4850919

[B3] Chandler J. (2011). The hormonal regulation of flower development. *J. Plant Growth Regul.* 30 242–254. 10.1007/s00344-010-9180-x

[B4] Chen C. Chen H. Zhang Y. Thomas H. R. Frank M. H. He Y. (2020). TBtools: an integrative toolkit developed for interactive analyses of big biological data. *Mol. Plant* 13 1194–1202. 10.1016/j.molp.2020.06.009 32585190

[B5] Edwards K. Fernandez-Pozo N. Drake-Stowe K. Humphry M. Evans A. Bombarely A. (2017). A reference genome for *Nicotiana tabacum* enables map-based cloning of homeologous loci implicated in nitrogen utilization efficiency. *BMC Genomics* 18:448. 10.1186/s12864-017-3791-6 28625162 PMC5474855

[B6] Estornell L. H. Wildhagen M. Pérez-Amador M. A. Talón M. Tadeo F. R. Butenko M. A. (2015). The IDA peptide controls abscission in Arabidopsis and Citrus. *Front. Plant Sci.* 6:1003. 10.3389/fpls.2015.01003 26635830 PMC4652038

[B7] Gubert C. M. Christy M. E. Ward D. L. Groner W. D. Liljegren S. J. (2014). ASYMMETRIC LEAVES1 regulates abscission zone placement in Arabidopsis flowers. *BMC Plant Biol.* 14:195. 10.1186/s12870-014-0195-5 25038814 PMC4223632

[B8] Hartmond U. Yuan R. Burns J. K. Grant A. Kender W. J. (2000). Citrus fruit abscission induced by methyl-jasmonate. *J. Am. Soc. Hortic. Sci.* 125 547–552. 10.21273/JASHS.125.5.547

[B9] Jinn T.-L. Stone J. M. Walker J. C. (2000). HAESA, an Arabidopsis leucine-rich repeat receptor kinase, controls floral organ abscission. *Genes Dev.* 14 108–117. 10.1101/gad.14.1.108 10640280 PMC316334

[B10] Katoh K. Standley D. M. (2013). MAFFT multiple sequence alignment software version 7: improvements in performance and usability. *Mol. Biol. Evol.* 30 772–780. 10.1093/molbev/mst010 23329690 PMC3603318

[B11] Kim J. Yang R. Chang C. Park Y. Tucker M. L. (2018). The root-knot nematode Meloidogyne incognita produces a functional mimic of the Arabidopsis INFLORESCENCE DEFICIENT IN ABSCISSION signaling peptide. *J. Exp. Bot.* 69 3009–3021. 10.1093/jxb/ery135 29648636 PMC5972575

[B12] Kumar S. Stecher G. Tamura K. (2016). MEGA7: molecular evolutionary genetics analysis version 7.0 for bigger datasets. *Mol. Biol. Evol.* 33 1870–1874. 10.1093/molbev/msw054 27004904 PMC8210823

[B13] Kumpf R. P. Shi C.-L. Larrieu A. Stø I. M. Butenko M. A. Péret B. (2013). Floral organ abscission peptide IDA and its HAE/HSL2 receptors control cell separation during lateral root emergence. *Proc. Natl. Acad. Sci. U. S. A.* 110 5235–5240. 10.1073/pnas.1210835110 23479623 PMC3612645

[B14] Lamesch P. Berardini T. Z. Li D. Swarbreck D. Wilks C. Sasidharan R. (2012). The Arabidopsis Information Resource (TAIR): improved gene annotation and new tools. *Nucleic Acids Res.* 40 D1202–D1210. 10.1093/nar/gkr1090 22140109 PMC3245047

[B15] Lescot M. Déhais P. Thijs G. Marchal K. Moreau Y. Van De Peer Y. (2002). PlantCARE, a database of plant cis-acting regulatory elements and a portal to tools for in silico analysis of promoter sequences. *Nucleic Acids Res.* 30 325–327. 10.1093/nar/30.1.325 11752327 PMC99092

[B16] Lewis M. W. Leslie M. E. Liljegren S. J. (2006). Plant separation: 50 ways to leave your mother. *Curr. Opin. Plant Biol.* 9 59–65. 10.1016/j.pbi.2005.11.009 16337172

[B17] Li X. Guo C. Ahmad S. Wang Q. Yu J. Liu C. (2019). Systematic analysis of MYB family genes in potato and their multiple roles in development and stress responses. *Biomolecules* 9:317. 10.3390/biom9080317 31366107 PMC6723670

[B18] Li X. Xing X. Tian P. Zhang M. Huo Z. Zhao K. (2018). Comparative transcriptome profiling reveals defense-related genes against *Meloidogyne incognita* invasion in tobacco. *Molecules* 23:2081. 10.3390/molecules23082081 30127271 PMC6222693

[B19] Li Z. Chao J. Li X. Li G. Song D. Guo Y. (2021). Systematic analysis of the bZIP family in tobacco and functional characterization of NtbZIP62 involvement in salt stress. *Agronomy* 11:148. 10.3390/agronomy11010148

[B20] Liljegren S. J. (2012). Organ abscission: exit strategies require signals and moving traffic. *Curr. Opin. Plant Biol.* 15 670–676. 10.1016/j.pbi.2012.09.012 23047135

[B21] Liu B. Butenko M. A. Shi C.-L. Bolivar J. L. Winge P. Stenvik G.-E. (2013). NEVERSHED and INFLORESCENCE DEFICIENT IN ABSCISSION are differentially required for cell expansion and cell separation during floral organ abscission in Arabidopsis thaliana. *J. Exp. Bot.* 64 5345–5357. 10.1093/jxb/ert232 23963677

[B22] Liu C. Zhang C. Fan M. Ma W. Chen M. Cai F. (2018). GmIDL2a and GmIDL4a, encoding the inflorescence deficient in abscission-like protein, are involved in soybean cell wall degradation during lateral root emergence. *Int. J. Mol. Sci.* 19:2262. 10.3390/ijms19082262 30072588 PMC6121880

[B23] Marciniak K. Kućko A. Wilmowicz E. Świdziński M. Przedniczek K. Kopcewicz J. (2018). Gibberellic acid affects the functioning of the flower abscission zone in Lupinus luteus via cooperation with the ethylene precursor independently of abscisic acid. *J. Plant Physiol.* 229 170–174. 10.1016/j.jplph.2018.07.014 30114566

[B24] Matsubayashi Y. Sakagami Y. (2006). Peptide hormones in plants. *Annu. Rev. Plant Biol.* 57 649–674. 10.1146/annurev.arplant.56.032604.144204 16669777

[B25] Meng D. Cao Y. Chen T. Abdullah M. Jin Q. Fan H. (2019). Evolution and functional divergence of MADS-box genes in Pyrus. *Sci. Rep.* 9:1266. 10.1038/s41598-018-37897-6 30718750 PMC6362034

[B26] Meng X. Zhou J. Tang J. Li B. De Oliveira M. V. Chai J. (2016). Ligand-induced receptor-like kinase complex regulates floral organ abscission in Arabidopsis. *Cell Rep.* 14 1330–1338. 10.1016/j.celrep.2016.01.023 26854226 PMC4758877

[B27] Mesejo C. Marzal A. Martínez-Fuentes A. Reig C. Agustí M. (2021). On how auxin, ethylene and IDA-peptide relate during mature Citrus fruit abscission. *Sci. Hortic.* 278:109855. 10.1016/j.scienta.2020.109855

[B28] Patharkar O. R. Walker J. C. (2018). Advances in abscission signaling. *J. Exp. Bot.* 69 733–740. 10.1093/jxb/erx256 28992277

[B29] Patharkar O. R. Walker J. C. (2019). Connections between abscission, dehiscence, pathogen defense, drought tolerance, and senescence. *Plant Sci.* 284 25–29. 10.1016/j.plantsci.2019.03.016 31084875

[B30] Patterson S. E. (2001). Cutting loose. Abscission and dehiscence in Arabidopsis. *Plant Physiol.* 126 494–500. 10.1104/pp.126.2.494 11402180 PMC1540116

[B31] Reichardt S. Piepho H.-P. Stintzi A. Schaller A. (2020). Peptide signaling for drought-induced tomato flower drop. *Science* 367 1482–1485. 10.1126/science.aaz5641 32217727

[B32] Santiago J. Brandt B. Wildhagen M. Hohmann U. Hothorn L. A. Butenko M. A. (2016). Mechanistic insight into a peptide hormone signaling complex mediating floral organ abscission. *Elife* 5:e15075. 10.7554/eLife.15075 27058169 PMC4848090

[B33] Schuster M. Van Der Hoorn R. A. (2020). Plant biology: distinct new players in processing peptide hormones during abscission. *Curr. Biol.* 30 R715–R717. 10.1016/j.cub.2020.04.072 32574636

[B34] Shi C.-L. Von Wangenheim D. Herrmann U. Wildhagen M. Kulik I. Kopf A. (2018). The dynamics of root cap sloughing in Arabidopsis is regulated by peptide signalling. *Nat. Plants* 4 596–604. 10.1038/s41477-018-0212-z 30061750

[B35] Stenvik G.-E. Butenko M. A. Aalen R. B. (2008a). Identification of a putative receptor-ligand pair controlling cell separation in plants. *Plant Signal. Behav.* 3 1109–1110. 10.4161/psb.3.12.7009 19704449 PMC2634470

[B36] Stenvik G.-E. Tandstad N. M. Guo Y. Shi C.-L. Kristiansen W. Holmgren A. (2008b). The EPIP peptide of INFLORESCENCE DEFICIENT IN ABSCISSION is sufficient to induce abscission in Arabidopsis through the receptor-like kinases HAESA and HAESA-LIKE2. *Plant Cell* 20 1805–1817. 10.1105/tpc.108.059139 18660431 PMC2518227

[B37] Tranbarger T. J. Domonhédo H. Cazemajor M. Dubreuil C. Fischer U. Morcillo F. (2019). The PIP peptide of INFLORESCENCE DEFICIENT IN ABSCISSION enhances Populus leaf and *Elaeis guineensis* fruit abscission. *Plants* 8:143. 10.3390/plants8060143 31151222 PMC6630328

[B38] Tucker M. L. Yang R. (2012). IDA-like gene expression in soybean and tomato leaf abscission and requirement for a diffusible stelar abscission signal. *AoB Plants* 2012:pls035. 10.1093/aobpla/pls035 23585923 PMC3624929

[B39] Vie A. K. Najafi J. Winge P. Cattan E. Wrzaczek M. Kangasjärvi J. (2017). The IDA-LIKE peptides IDL6 and IDL7 are negative modulators of stress responses in Arabidopsis thaliana. *J. Exp. Bot.* 68 3557–3571. 10.1093/jxb/erx168 28586470 PMC5853212

[B40] Wang R. Shi C. Wang X. Li R. Meng Y. Cheng L. (2020). Tomato SlIDA has a critical role in tomato fertilization by modifying reactive oxygen species homeostasis. *Plant J.* 103 2100–2118. 10.1111/tpj.14886 32573872

[B41] Wang X. Hou S. Wu Q. Lin M. Acharya B. R. Wu D. (2017). IDL6-HAE/HSL2 impacts pectin degradation and resistance to *Pseudomonas* syringae pv tomato DC3000 in Arabidopsis leaves. *Plant J.* 89 250–263. 10.1111/tpj.13380 27618493

[B42] Wilmowicz E. Kućko A. Ostrowski M. Panek K. (2018). INFLORESCENCE DEFICIENT IN ABSCISSION-like is an abscission-associated and phytohormone-regulated gene in flower separation of *Lupinus luteus*. *Plant Growth Regul.* 85 91–100. 10.1007/s10725-018-0375-7

[B43] Ying P. Li C. Liu X. Xia R. Zhao M. Li J. (2016). Identification and molecular characterization of an IDA-like gene from litchi, LcIDL1, whose ectopic expression promotes floral organ abscission in Arabidopsis. *Sci. Rep.* 6:37135. 27845425 10.1038/srep37135 PMC5109030

[B44] Zhang X. Peng H. Zhu S. Xing J. Li X. Zhu Z. (2020). Nematode-encoded RALF peptide mimics facilitate parasitism of plants through the FERONIA receptor kinase. *Mol. Plant* 13 1434–1454. 10.1016/j.molp.2020.08.014 32896643

