# Supplementary figures and images for: Bioinformatics and Expression Analysis of IDA-Like Genes Reveal Their Potential Functions in Flower Abscission and Stress Response in Tobacco (Nicotiana tabacum L.)
Source: Front Genet. 2021 Apr 27;12:670794. doi: 10.3389/fgene.2021.670794 (PMC8110903; doi:10.3389/fgene.2021.670794)

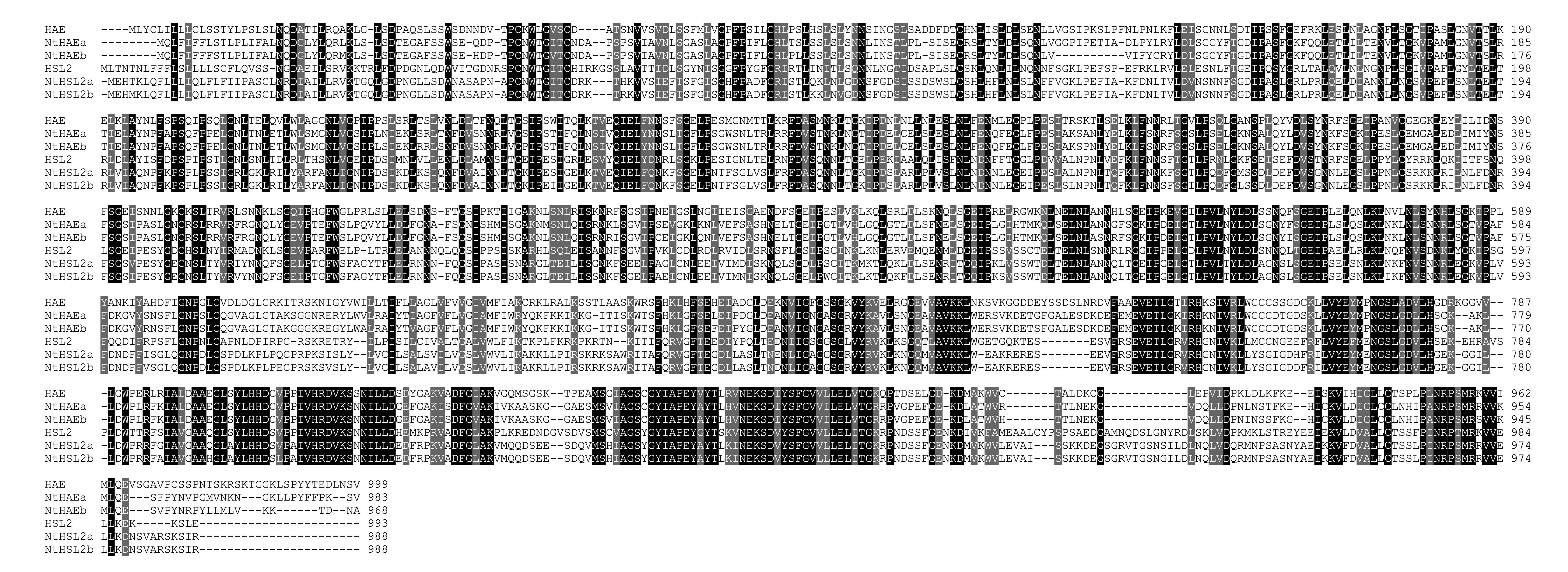

Supplement: Supplementary Figure 1 — Sequence alignment of HAE-Like proteins from tobacco and Arabidopsis. The black background indicates that the amino acid similarity is 100%, and the gray indicates that the amino acid similarity is ranged from 60 to 90%. [file Image_1.JPEG]

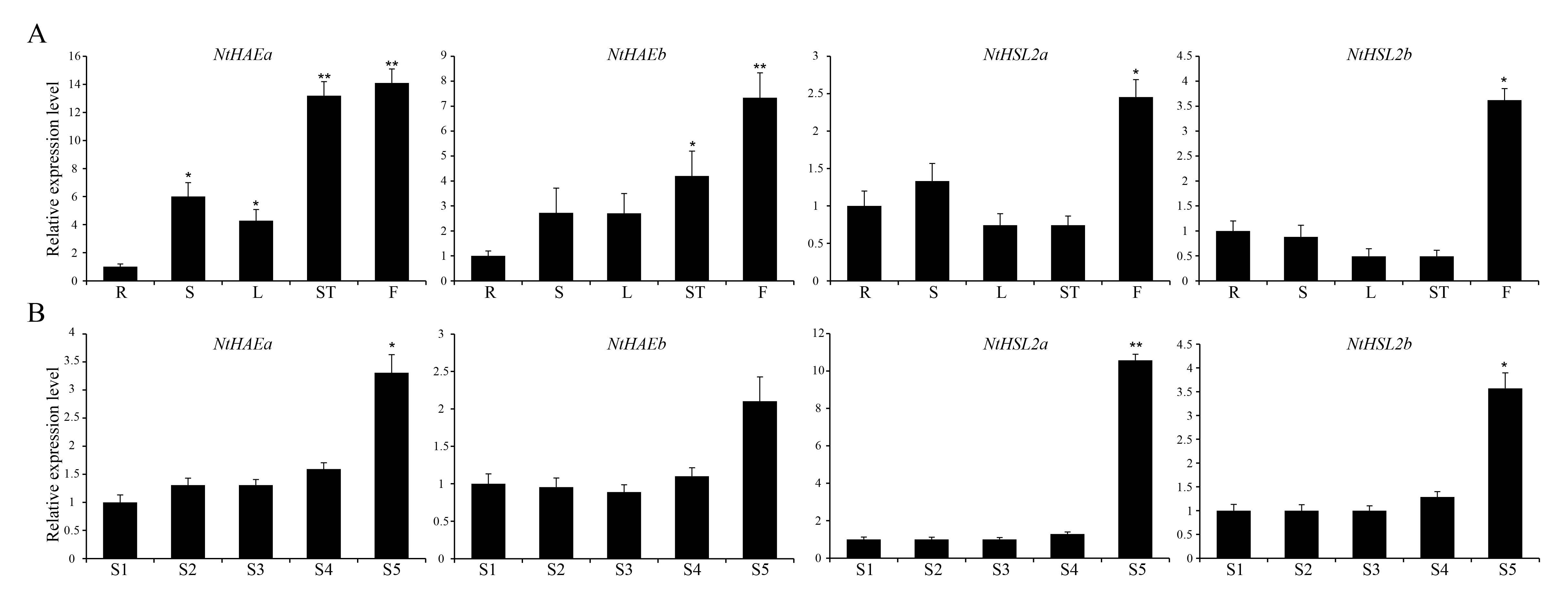

Supplement: Supplementary Figure 2 — The expression pattern of NtHAE-Like genes in selected tissues (A) and in abscission zone during the flower development (B). Panel (A): “R,” “S,” “L,” “ST,” and “F” mean roots, stems, leaves, shoots and flowers, respectively. Their expressions were calculated as folds relative to the expression level of the roots to confirm the tissue specificity. Panel (B): “S1–S5” means “Stage 1 to Stage 5 of flower development.” All expression levels were calculated through the 2–ΔΔCt method. The data were means ± SD from three independent replications. *p < 0.05, **p < 0.01, (t-test). [file Image_2.JPEG]
